# Steroid psychosis in a polyarteritis nodosa patient successfully treated with risperidone: tracking serum brain-derived neurotrophic factor levels longitudinally

**DOI:** 10.1186/1744-859X-11-2

**Published:** 2012-01-23

**Authors:** Reiji Yoshimura, Kosuke Saito, Tadanori Terada, Naoki Yunoue, Wakako Umene-Nakano, Shintaro Hirata, Kazuyoshi Saitoh, Yoshiya Tanaka, Jun Nakamura

**Affiliations:** 1 Department of Psychiatry, University of Occupational and Environmental Health, Iseigaoka, Yahatanishi-ku, Kitakyushu, Fukuoka 8078555, Japan; 2 Department of Anesthesiology, University of Occupational and Environmental Health, Iseigaoka, Yahatanishi-ku, Kitakyushu, Fukuoka 8078555, Japan; 3 First Department of Internal Medicine, University of Occupational and Environmental Health, Iseigaoka, Yahatanishi-ku, Kitakyushu, Fukuoka 8078555, Japan

## Abstract

We previously reported a case in which steroid-induced psychosis was eliminated with risperidone treatment in a patient with polyarteritis nodosa (PN). In the present report, we longitudinally tracked the serum levels of brain-derived neurotrophic factor (BDNF). We found that corticosteroid lowered serum BDNF levels, and improvement of psychiatric symptoms was intact with the serum BDNF levels seen in the patients.

## Introduction

There are several reports demonstrating the effectiveness of risperidone in treating steroid psychosis [1,2]. We have also previously demonstrated that risperidone did not change serum brain-derived neurotrophic factor (BDNF) levels in patients with schizophrenia [3]. BDNF is associated with psychiatric diseases such as depression or schizophrenia [1]. In the present case, risperidone rapidly diminished our patient's psychiatric symptoms without severe adverse effects. Corticosteroids suppress BDNF levels in the brain, which leads to atrophy of the hippocampus [4]. To the best of our knowledge, this is the first report showing longitudinal tracking of serum BDNF levels in a case of steroid psychosis in a patient with polyarteritis nodosa (PN).

## Case presentation

**Figure 1:**
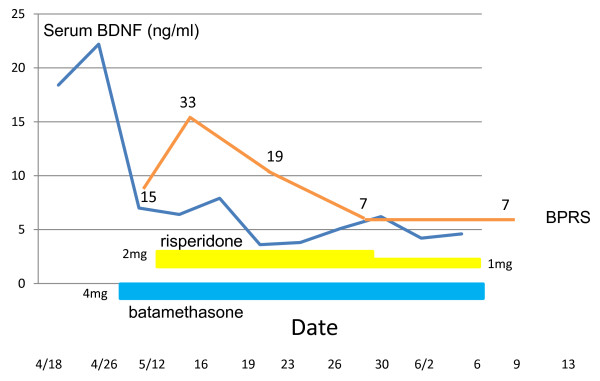
**Changes in serum brain-derived neurotrophic factor (BDNF) levels and Brief Psychiatric Rating Scale (BPRS) scores**.

Our patient, a 69-year-old woman, had had a diagnosis of PN for 4 years with no previous psychiatric history. Her major symptoms of PN were hypertension, pleuritis, vasculitis, a raised platelet count, and a high level of C-reactive protein. She did not have positive findings for vasculitis in the brain on MRI or magnetic resonance angiography (MRA). She had been treated with steroid pulse therapy (intravenous administration of methylprednisolone at 45 mg/day) followed by betamethasone at 4 mg/day. After 1 month at this dosage, she had experienced a mixed state that included being more talkative than usual, feeling hyperactive, and excited, crying, feeling depressed, and having rapid mood swings, persecutory delusions, and auditory hallucinations. Her score on the Brief Psychiatric Rating Scale (BPRS) was 33 points. Risperidone was started at 1 mg/day and increased to 2 mg/day, and the dose of betamethasone was continued at the same dose (4 mg/day). Her psychiatric symptoms gradually improved, and she reached remission 3 weeks after the initiation of risperidone treatment. Since she demonstrated mild finger tremor, her dose of risperidone was decreased to 1 mg/day without worsening of her psychiatric symptoms. During her course of psychiatric symptoms, we longitudinally measured her serum BDNF levels, as shown in Figure 1.

## Conclusions

Corticosteroid reduced serum BDNF levels and kept those levels lower, and risperidone did not cause the serum BDNF levels to recover. In addition, risperidone is effective for use in steroid psychosis patients with PN, and improvement of psychotic symptoms in the patient was independent of serum BDNF levels.

## Consent

Written informed consent was obtained from the patient for publication of this case report.

## Competing interests

The authors declare that they have no competing interests.

## Authors' contributions

RY, KS, TT, NY and SH were crucially involved in the treatment process described for our patient. WU-N assayed serum BDNF levels. KS, YT and JN critically revised the manuscript and gave their final approval for the version to be published.
